# Low Mood Leads to Increased Empathic Distress at Seeing Others’ Pain

**DOI:** 10.3389/fpsyg.2017.02024

**Published:** 2017-11-20

**Authors:** Yuan Cao, Genevieve Dingle, Gary C. K. Chan, Ross Cunnington

**Affiliations:** ^1^School of Psychology, University of Queensland, Brisbane, QLD, Australia; ^2^Centre for Youth Substance Abuse Research, Royal Brisbane and Women’s Hospital, Brisbane, QLD, Australia; ^3^Queensland Brain Institute, University of Queensland, Brisbane, QLD, Australia

**Keywords:** empathy, personal distress, depression, sadness, mood induction

## Abstract

Previous studies have shown changes in empathy in patients with depression, including an elevated level of trait personal distress. This study examined if low mood causes changes in self-reported empathic distress when seeing others in pain. To test this, we conducted an initial (*n* = 26) and close replication study (*n* = 46) in which sad mood was induced in healthy participants (overall mean age *M* = 21, *SD* = 5, range = 18–41 years). Participants viewed and rated video stimuli inferring pain experienced by other people. Results showed that participants perceived the videos depicting others’ pain (versus no-pain) to be more distressing under a sad mood compared to a neutral mood condition, implying that sadness enhances one’s emotional reactivity toward others’ distress. This supports previous depression literature suggesting an impaired emotional processing ability, and could contribute to some of the unhelpful behaviors seen in depression such as social withdrawal and avoidance.

## Introduction

Empathy is an important aspect of social interactions as it promotes prosocial behaviors (Batson and Shaw, 1991; Baron-Cohen and Wheelwright, 2004; De Waal, 2008), cooperation and forgiveness (Xu et al., 2012; Ricciardi et al., 2013), and affects the quality of close relationships (Coutinho et al., 2014). However, it also constitutes emotional distress for the empathizer or “personal distress” (Davis, 1980). Although sometimes empathic personal distress might contribute to better friendship quality, it is also related to higher anxiety levels for the empathizer (Smith, 2015). Personal discomfort relating to other’s distress is also suggested to foster a sense of guilt for the empathizer and lead to behaviors such as avoidance or over-costly helping behaviors (O’Connor et al., 2012). Although people with clinical depression appear to experience this negative side of empathy (Schreiter et al., 2013), which has been proposed to contribute to the excessive guilt that is commonly seen in depression (O’Connor et al., 2012), there is little (if any) experimental research examining the nature of the relationship between personal distress and depression. To better understand the relationship between empathic distress and low mood specifically, as a major aspect of depression, the current study utilizes a mood induction paradigm to examine such relationship in healthy participants.

Mood induction is used in the current study to experimentally change healthy participants’ mood temporarily, in order to examine the effect of induced sadness on empathic personal distress. Mood induction has been used previously to study the effect of mood instead of examining clinically depressed participants, as it allows for better control of confounding variables, such as presence of comorbid disorders and use of medications (Berna et al., 2010; Robinson et al., 2012). It has been shown that mood induction simulates behavioral changes that are similar to some aspects of depression, in addition to self-reported mood changes, including changes in speed on behavioral tasks, presence of pauses during communication, non-verbal communication, and reduced persistence (Goodwin and Williams, 1982).

Mood induction methods have been used in previous studies examining the effect of mood on first-hand pain. It appears that positive affect decreases pain perception, whereas negative affect may increase pain perception, although the later effect seems to be less consistent across studies (Villemure and Bushnell, 2002). For example, by enhancing participants’ mood through delivery of pleasant odors, Villemure and Bushnell (2009) reported modulation in the activity of the pain network when the participants received heat stimuli. Furthermore, mood induction has also been used in the pain literature to show that low mood leads to increased unpleasantness of experienced pain (Berna et al., 2010). Using a combination of sad music and negative statements, Berna et al. (2010) studied the effect of depressive mood on neural processing of first-hand pain. Results showed that participants responded more strongly to the physical pain that they experienced when feeling sad, and areas such as the subgenual part of the anterior cingulate cortex showed more activation in the depressive condition compared to the neutral condition. As their mood induction paradigm successfully lowered people’s mood, which then impacted their experience of first-hand pain, a modified version of their mood induction paradigm is employed for the current study to examine the direct effect of change in mood (namely, low mood) on empathic distress for others’ pain. The hypotheses of the current study were formed based on previous findings from clinical studies.

In survey studies, trait empathy has been commonly examined using the self-report questionnaire Interpersonal Reactivity Index (IRI; Davis, 1980). People with depression have repeatedly scored higher than healthy control groups on the Personal Distress subscale of the IRI, which suggests that they tend to experience more distress upon seeing others in emotional situations such as in emergencies (O’Connor et al., 2002; Thoma et al., 2011; Schreiter et al., 2013). More recently, Tully et al. (2015) reported that participants who had elevated empathic concern and poor emotion regulation were also more likely to experience depression.

Behavioral studies have examined state empathy in depression, focusing on cognitive empathy, or cognitive evaluations of others’ emotional states, and they report conflicting results. Derntl et al. (2012) found that compared to the healthy controls, participants with depression had deficits in correctly imagining the emotion one would be in from reading and relating to situations described in the stimuli. Harkness et al. (2010) reported enhanced theory of mind in remitted depression compared to healthy controls, while others found no difference between patients in remission from clinical depression and a healthy control sample on a basic theory of mind task (Inoue et al., 2004). It appears that only a few studies have examined state emotional empathy in depression using behavioral measures. Depressed patients were less accurate on labeling the other person’s feelings in Schneider et al. (2012) study, and had fewer feelings that matched with that of the actors in the videos. Yet the depressed patients had heightened galvanic skin conductance responses to the videos, reflecting a higher level of physiological arousal in response to the emotional task. More recently, Hoffmann et al. (2016) demonstrated increased emotional contagion in depression, as the depressed participants’ rating of the pleasantness of tactile stimuli that were delivered to them was affected by what they observed to be delivered to another participant at the same time.

Empathy has also been an area of interest in neuroscience, particularly focusing on empathy for pain (e.g., Xu et al., 2009; Fan et al., 2011; Contreras-Huerta et al., 2013; Cao et al., 2015). Analysis is typically done by contrasting or subtracting responses to a control or non-painful stimuli from that to the painful stimuli, resulting in the so called neural empathy for pain network. In this paradigm, increased activation in the medial portion of the cingulate cortex and the anterior insula cortex have been repeatedly reported (Lamm et al., 2011). Existing data from fMRI studies suggests that activity in the cingulate cortex in response to emotional stimuli is affected by depression (Fujino et al., 2014; Merkl et al., 2015; Regenbogen et al., 2015).

In summary, the available questionnaire studies demonstrate that depression is accompanied by a heightened personal distress and altered cingulate activity at seeing others’ suffering. However, it is unclear if there is a direct and casual relationship between depression and exaggerated empathic distress. Also, there is relatively limited behavioral research on state emotional empathy in low mood and depression that involves an indicator of the observer’s own experience. As a first step in better understanding the relationship between mood state and empathic distress, the current study examines causal relationships between low mood and heightened empathic distress at seeing others’ pain in healthy participants. As explained above, mood induction in healthy participants could assist us in focusing specifically on the low mood component that is also prominent in depression, without the other potential confounding variables such as symptom complexity and medication effects. To the best of our knowledge, this is the first study to examine if there is a causal relationship between low mood and heightened personal distress.

To conclude, the current study aimed to establish a causal relationship between sad mood and increased personal distress at seeing another’s pain in healthy participants. It was hypothesized that there would be a larger difference between distress ratings for painful videos relative to non-painful videos under sad mood compared to neutral mood.

## Materials and Methods

### Participants

Caucasian volunteers without any self-reported current or previous psychiatric or neurological issues were recruited through online advertisements. Only Caucasian volunteers were recruited to limit any potential racial effect when viewing the video stimuli of Caucasian actors (Xu et al., 2009). Participants also passed a screening measure for depression, anxiety and stress, using the Depression Anxiety Stress Scale (DASS; Lovibond and Lovibond, 1995), as it has been found that stress can have a negative effect on pain perception (Buruck et al., 2014) and empathy (Martin et al., 2015). In total, 87 people were recruited over two stages of the study, and 15 were excluded due to ineligibility (10 due to stress, anxiety, or depression above the Normal range on the DASS, one due to current psychiatric issues, and four withdrew from the study before the second session).

The study was conducted in two parts, comprising an initial study and a follow-up close replication study in independent groups of healthy participants. In the first study, 26 participants passed the screening and completed the experimental session (14 males; age *M* = 20.54, *SD* = 3.09, range = 18–30). Although not included in the eligibility criteria, most participants had some exposure to music in school (50% had secondary school musical lessons, 30.8% had only primary school music classes, 19.2% had none). Although initial statistical analysis (using repeated-measures ANOVA) showed a significant mood × video interaction effect, there was a risk of false positive results with a small sample size (Colquhoun, 2014). Hence, we conducted a replication study using the same recruitment and experimental procedures. Power analysis based on the initial results from the initial study indicated that a sample size of 46 would be required to achieve power of 0.9 (dependent means *t*-test, G-Power, based on effect size dz = 0.49 [from the initial study sample] and alpha = 0.05). Therefore, in the replication study, 46 participants were recruited, and they all passed the screening and completed the experimental session (23 males; age *M* = 21.70, *SD* = 6.17, range = 18–41), again with most having had exposure to music in school (2.2% had tertiary classes, 41.3% had secondary school lessons, 45.7% had only primary school music classes, and 10.9% had none).

This study was reviewed and approved by the Behavioural and Social Sciences Ethical Review Committee at the University of Queensland and was conducted with written informed consent from all participants.

### Procedure

#### Session 1: Screening

There were two sessions to the study. In the first, participants completed a screening process to ensure suitability, which involved completing the DASS (Lovibond and Lovibond, 1995). Participants’ level of musical training was gathered through the Music USE Questionnaire (Chin and Rickard, 2012). Participants also completed other measures related to music that were not included in the current study.

#### Session 2: Mood Induction and Empathic Distress

In session 2, each participant received two mood inductions, a sad mood induction and a neutral mood induction (Berna et al., 2010; order was counterbalanced). Each induction was followed by an empathy for pain task that involved watching videos of people receiving painful or non-painful touch and giving ratings on their experienced distress in response to each video (adapted based on Cao et al., 2015).

During the 6-min mood induction, participants read statements on a computer display while listening to mood-coherent classical music through headphones. For the sad mood condition, participants listened to Prokofiev’s “Russia Under the Mongolian Yoke” at half speed while reading statements such as “There is no hope,” “I am tired of trying,” and “Life is such a heavy burden.” For the control condition, which was a neutral mood condition, Dvorak’s “Symphony from the New World” was played at its usual speed while participants saw statements such as “It snows in Scotland,” “An orange is a citrus fruit,” and “The Pacific Ocean has fish.” Interested readers could email the authors for a copy of the statements used in the mood inductions. Participants rated their mood prior to and after each mood induction on a scale from 0 = not at all to 10 = extremely for two items: “At this moment I feel SAD” and “At this moment I feel HAPPY.” To help maintain the induced mood, the mood-congruent music continued to play after the induction throughout the following empathy task. Participants rated their mood (on both the sadness and happiness questions) again on the 11-point scale after the empathy task, which was also the end of that mood condition.

For the empathy task, immediately after completing each of the mood inductions, participants watched videos of Caucasian actors receiving painful (with a syringe needle) and non-painful (with a cotton-tip) touches on the cheek. A total of 32 videos were shown, portraying all combinations of eight actors (four males and four females) receiving a painful or non-painful touch to the left or the right cheek of their face (3-s videos, 9 s inter-stimulus interval). To limit emotional contagion, the video clips were cut at the moment that the needle or the cotton-tip made contact with the cheek, so that participants did not observe emotional expressions of the actors in response to the touches. Participants were asked to rate how distressing they found watching each of the videos, by pressing one of the five keys on a keyboard (1 = not distressing at all to 5 = very distressing). There were a total of four blocks of this empathy task, two for each mood condition. A 6-min break time was scheduled after two blocks, during which the participant was told to relax and listen to a new piece of music. A positive piece of classical music, Delibes’ “Coppelia” (chosen based on Vastfjall, 2002) was played after the first mood induction, both for neutral and sad mood conditions.

### Data Analysis

The analyses were carried out using SPSS 24 and the focus was on the participant’s responses to the mood induction paradigm, and ratings for the video stimuli on the empathy task.

A sad mood composite score was calculated using the formula {[(10-happy)+sad]/2} (Berna et al., 2010). Therefore, the higher the score, the sadder and/or less happy the participant felt. To check the validity of the mood induction, repeated measures ANOVA analyses were conducted on the mood composite score in the two mood conditions (sad/neutral) at three time points (prior to and immediately after mood induction, and on conclusion of that mood condition, which was when a round of the empathy task finished). Follow up *t*-tests with Bonferroni correction were then performed. There were two missing values at the end of the mood conditions for sample 1, as one participant did not give mood ratings at the end of the neutral mood condition, and another participant did not give mood ratings after the sad mood condition. To ensure that participants’ mood did not change significantly in the neutral mood condition, their happiness and sadness ratings were compared from before to after the neutral mood induction, using Wilcoxon Signed Ranks Tests. This is because the individual sadness ratings were sometimes highly positively skewed.

A mean distress rating was calculated from the participants’ scores on the five-point scale for each of the two types of videos (painful or non-painful) under each mood condition (sad or neutral). For participants with missing responses, the mean distress rating was calculated based on the available responses. Two participants had more than 10% responses missing due to technical issues during testing (14 and 27% responses missing, respectively), and their mean responses were included in the analysis.

Overall, the distress rating data were found to be highly skewed, due to low scores repeatedly given to the cotton-tip videos (more than 40% of the mean ratings given to the cotton-tip videos were ‘1’ or not distressing at all). Therefore, non-parametric methods were utilized to analyze the distress ratings. It should be noted that our initial analyses on the first sample of participants were conducted using repeated-measures ANOVA; however, on inspection of the skewness of the data (and following comments from an external referee), we decided on more conservative non-parametric analyses for the distress rating data across both the initial and replication studies.

For non-parametric analysis of the distress ratings, therefore, a Friedman Test was used first to examine whether the ratings were significantly different between the four conditions (painful in sad condition, non-painful in sad condition, and painful and non-painful in neutral condition). Follow up pairwise comparisons between conditions were then conducted using Wilcoxon Signed Rank Tests. Effect sizes were estimated with r using the formula of the absolute value of z divided by the square root of n (number of observations; Pallant, 2013).

To further examine differences in distress ratings for painful relative to non-painful videos between the two mood conditions, a difference score was calculated between the distress rating given to the painful videos and that given to the non-painful videos, under each of the two mood conditions (i.e., pain minus non-pain for sad, compared with pain minus non-pain for neutral). These empathic distress difference scores were compared between mood conditions using a Wilcoxon Signed Rank Test. As consent for data sharing was not obtained from participants for this study, the individual data is not made publically available.

## Results

### Initial Study: Participant Sample One

#### Mood Induction

**FIGURE 1:**
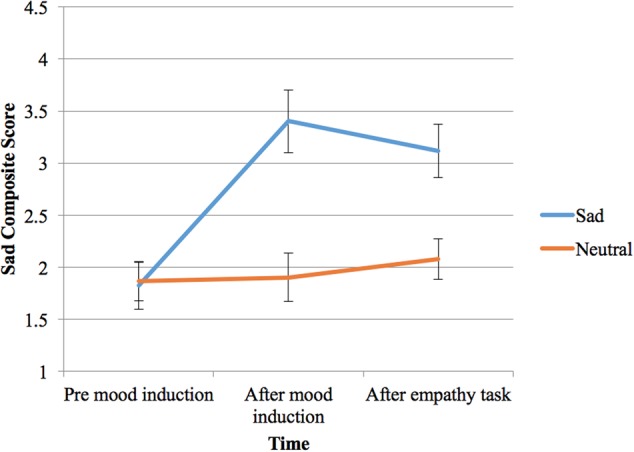
Participant sample one’s self-reported mood (composite score from 0 to 10, 0 = not sad at all and extremely happy; 10 = not happy at all and extremely sad) before and after two types of mood induction (sad or neutral), and at the end of the empathy task (bars shown are standard errors).

Participants’ responses to the mood induction, or their sad composite scores, were affected by the type of mood induction, *F*(1,23) = 47.57, *p* < 0.001, $\eta_{p}^{2}$= 0.67, the time since mood induction, *F*(2,46) = 22.97, *p* < 0.001, $\eta_{p}^{2}$= 0.50, and an interaction of these two factors, *F*(2,46) = 19.72, *p* < 0.001, $\eta_{p}^{2}$= 0.46 (see **Figure 1**).

Importantly, the sad composite score was significantly greater both following the sad mood induction (*p* < 0.001) and after the empathy task (*p* < 0.001) compared with the initial score prior to induction. On the other hand, the sad composite score did not change significantly from before to after the neutral mood induction, or at the end of the neutral condition (*p* > 0.05).

Most importantly, comparing neutral and sad mood induction conditions, participants’ sad composite scores were not significantly different before mood induction, but were significantly higher for the sad induction both immediately after mood induction and after the empathy task (both *p* < 0.001). Therefore, the sad induction successfully elicited more sad feelings in the participants, compared to before induction and compared to the neutral condition.

#### Empathy Task: Ratings of Video Stimuli

**FIGURE 2:**
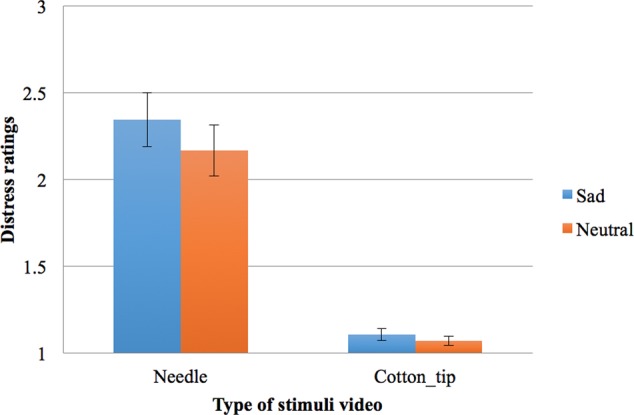
Participant sample one’s distress ratings on a scale from 1 to 5 (1 = not distressing at all, 5 = very distressing) for two types of stimuli (painful needles or non-painful cotton-tips) in the two mood conditions (bars shown are standard errors).

Friedman test indicated that the rankings of the four distress ratings (given to the two types of videos under two mood conditions) varied significantly, $\chi_{F}^{2}$ = 63.54, *df* = 3, *N* = 26, *p* < 0.001 (see **Figure 2**).

Follow-up pairwise comparisons with the Wilcoxon Signed Rank test and a Bonferroni adjusted α of 0.013 indicated that the needle videos were rated as more distressing under sad mood (*Mean Rank* = 3.71) than neutral mood (*Mean Rank* = 3.13), *T* = 221.50, *z* = -2.54, *N* = 26, *p* = 0.011, *r* = 0.35. On the other hand, the ratings given to the cotton-tip videos did not differ significantly from the sad condition (*Mean Rank* = 1.75) to the neutral condition (*Mean Rank* = 1.40, *T* = 64, *z* = -1.96, *p* = 0.05 without Bonferroni correction, *r* = 0.27). As expected, the needle videos were rated as more distressing than the cotton-tip ones under both mood conditions, under sad mood, *T* = 325, *z* = -4.37, *p* < 0.001, *r* = 0.61, and under neutral mood, *T* = 300, *z* = -4.29, *p* < 0.001, *r* = 0.59.

To further examine if the change in distress rating (painful vs. non-painful touch) was different across the two mood conditions, two difference scores were calculated for the distress rating given to the needle videos minus that given to the cotton-tip, under each mood condition. These difference scores represent the participant’s level of empathic distress, i.e., how much more distressing the participant experienced the needle videos relative to the cotton-tip videos. These empathic distress scores were marginally higher under sad mood compared with neutral mood, according to Wilcoxon Signed Rank test, *T* = 202, *z* = -1.95, *p* = 0.052, *r* = 0.27.

### Participant Sample Two

#### Mood Induction

**FIGURE 3:**
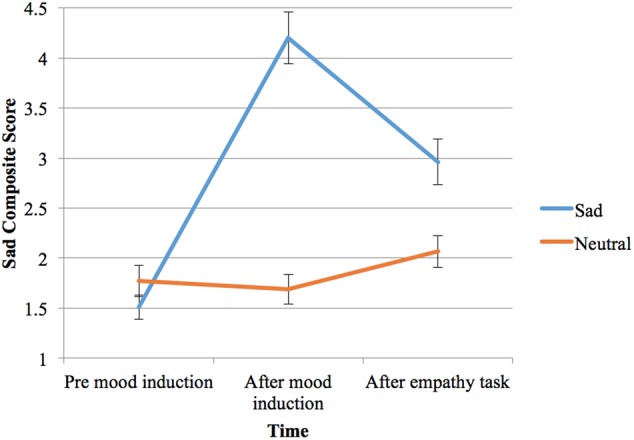
Participant sample two’s self-reported mood (composite score from 0 to 10, 0 = not sad at all and extremely happy; 10 = not happy at all and extremely sad) before and after two types of mood induction (sad or neutral), and at the end of the empathy task (bars shown are standard errors).

Participants’ responses to the mood induction, or their sad composite scores, were affected by the type of mood induction, *F*(1,45) = 83.68, *p* < 0.001, $\eta_{p}^{2}$= 0.65, the time since mood induction, *F*(2,90) = 71.56, *p* < 0.001, $\eta_{p}^{2}$= 0.61, and an interaction of these two factors, *F*(2,90) = 74.87, *p* < 0.001, $\eta_{p}^{2}$= 0.63 (see **Figure 3**).

Importantly, the sad composite score was significantly greater both following the sad mood induction (*p* < 0.001) and after the empathy task (*p* < 0.001) compared with the initial score prior to induction. On the other hand, the sad composite score did not change significantly from before to after the neutral mood induction, although it increased slightly at the end of the neutral condition (from 1.69 to 2.07, *p* = 0.001), possibly due to the relatively repetitive empathy task.

Most importantly, comparing neutral and sad mood induction conditions, participants’ sad composite scores were not significantly different before mood induction, but were significantly higher for the sad induction both immediately after mood induction and after the empathy task (both *p* < 0.001). Therefore, the sad induction successfully elicited more sad feelings in the participants, compared to before induction and compared to the neutral condition.

#### Empathy Task: Ratings of Video Stimuli

**FIGURE 4:**
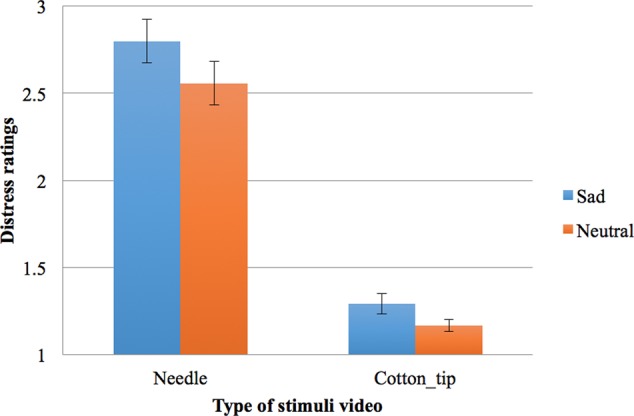
Participant sample two’s distress ratings on a scale from 1 to 5 (1 = not distressing at all, 5 = very distressing) for two types of stimuli (painful needles or non-painful cotton-tips) in the two mood conditions (bars shown are standard errors).

Friedman test indicated that the rankings of the four distress ratings (given to the two types of videos under two mood conditions) varied significantly, $\chi_{F}^{2}$ = 111.71, *df* = 3, *N* = 46, *p* < 0.001 (see **Figure 4**).

Follow-up pairwise comparisons with the Wilcoxon Signed Rank test and a Bonferroni adjusted α of 0.013 were conducted. As expected, the needle videos were rated as more distressing than the cotton-tip videos under both mood conditions (sad mood, *T* = 990, *z* = -5.78, *p* < 0.001, *r* = 0.60; neutral mood, *T* = 1035, *z* = -5.84, *p* < 0.001, *r* = 0.61). In addition, results indicated that the needle videos were rated as more distressing under sad mood (*Mean Rank* = 3.72) than neutral mood (*Mean Rank* = 3.18), *T* = 844, *z* = -4.07, *N* = 46, *p* < 0.001, *r* = 0.42. The ratings given to the cotton-tip videos also differed significantly from the sad condition (*Mean Rank* = 1.71) to the neutral condition (*Mean Rank* = 1.39, *T* = 433.50, *z* = -2.74, *p* = 0.006 without Bonferroni correction, *r* = 0.29).

To further examine if the change in distress rating (painful vs. non-painful touch) was different across the two mood conditions, two difference scores were calculated for the distress rating given to the needle videos minus that given to the cotton-tip, under each mood condition. These difference scores represent the participant’s level of empathic distress, i.e., how much more distressing the participant experienced the needle videos relative to the cotton-tip videos. Crucially, these empathic distress scores were significantly higher under sad mood compared with neutral mood, according to Wilcoxon Signed Rank test, *T* = 691, *z* = -2.29, *p* = 0.022, *r* = 0.24. Therefore, the difference in the distress ratings for the needle compared with the cotton-tip videos was significantly larger in the sad mood condition than the neutral condition, reflecting a relative increase in empathic distress with sad mood.

### *Post hoc* Analysis: Order of Mood Induction

A *post hoc* test was conducted to examine any possible effect of the order of the mood induction (i.e., sad followed by neutral versus neutral followed by sad). This *post hoc* analysis was pooled across the full sample of 72 participants, across both the initial and replication studies. Specifically, we examined whether the sad mood composite score was different prior to neutral mood induction for participants who received the sad induction first compared with those who received it second. It was found that those who had sad induction first did have a higher sad mood composite score immediately prior to the neutral mood induction as compared with those who had the neutral condition first, *t*(70) = 2.66, *p* = 0.01. Crucially, however, the two groups did not differ significantly in their sad mood composite score at the time after the neutral mood induction immediately before doing the empathy task in neutral mood (*p* = 0.29). Therefore, it appears that sad mood may have lasted some time following completion of the empathy task in the sad condition (contributing to differences immediately before neutral mood induction in those participants who completed the sad condition first); however, crucially all participants had similar mood immediately following the neutral mood induction when completing the empathy task in the neutral mood condition.

## Discussion

Extending previous survey findings of a relationship between low mood and empathy (Schreiter et al., 2013), this study directly manipulated mood in healthy participants to examine whether low mood causes changes in self-reported empathic distress upon seeing stimuli inferring others’ pain. Indeed, it was found that participants perceived stimuli depicting a painful touch to another person to be more distressing under sad mood compared to a neutral mood condition. Crucially, the participants’ level of empathic distress, defined as how much more distressing they experienced the observed painful touch than the non-painful touch, was significantly greater under sad mood than neutral mood. This finding is consistent with previous clinical literature showing a correlation between depression and higher trait personal distress (O’Connor et al., 2002; Thoma et al., 2011), and is in line with Decety and Lamm’s (2009) model that empathy can entail the feeling of distress when observing another’s difficult situation. The current finding extends on previous literature by showing the *causal* effect of momentary low mood on increasing empathic distress to others’ pain.

It has been proposed that higher empathic distress contributes to guilt, which in turn makes depression more likely or contributes to its maintenance (O’Connor et al., 2012). The current study showed that a low mood state causes greater empathic distress at seeing others’ pain. This, in combination with previous findings from depressed patients as discussed above (O’Connor et al., 2002; Thoma et al., 2011), appears in line with the idea that heightened empathic distress to others’ suffering is an ongoing issue in depression. This is problematic as the distress at seeing others’ negative situations may lead to some of the unhelpful behaviors seen in depression such as social withdrawal or avoidance (Seidel et al., 2010; O’Connor et al., 2012). Therefore, it may be useful to conduct similar studies on people at risk of developing depression, to see whether their empathic distress responses are further exaggerated under low mood.

It may be noted that the difference between the distress ratings for painful videos relative to non-painful videos under sad mood compared to neutral mood was statistically significant in the second sample, but was not significant in the first sample. This is likely to be due to the limited power associated with the sample size of the first group. The first group did show a trend for the difference score to be larger in the sad condition and, importantly, the effect sizes for this difference were similar in both the first and second samples (*r* = 0.27 and *r* = 0.24 respectively). Therefore, although the first study failed to detect a statistically significant difference, the overall pattern of results was the same across both sample groups, with the needle videos relative to the cotton-tip videos perceived to be more distressing under sad mood than neutral mood.

Also, it should be noted that the participants’ distress ratings given to the cotton-tip videos also increased following the sad mood induction, although the size of this effect was smaller than for the painful needle videos. One possibility is that this may reflect a heightened sensitivity toward these types of personal touch stimuli in general under low mood. While designed to appear neutral, and clearly rated by participants as less distressing than the needle-touch videos, the cotton-tip touch as depicted in the videos was not particularly pleasant or pleasurable and may therefore have been perceived as slightly more unpleasant or distressing for participants under low mood. Crucially, however, even when controlling for this difference in “baseline” distress ratings to cotton-tip videos with mood (i.e., by examining the relative difference between painful and non-painful touch), the increase in distress ratings seen for needle versus cotton-tip videos was significantly greater under sad mood than neutral mood.

A potential limitation of the current study was the playing of music during the empathy task, which is an adaptation of the original mood induction paradigm by Berna et al. (2010). The intention was to help with sustaining the low mood induced prior to the empathy task, with minimum interruption to the visual empathy task. However, as the main results as discussed above are based on comparisons between the sad mood and the neutral mood conditions that equally involved music, it is unlikely that the sad mood effect is fully explained by the mere presence of music during the empathy task. However, to fully separate the effect of mood from the potential confound of music, an alternate adaptation of the current mood induction paradigm, without the need for continued music, would be helpful to explore in future research.

In summary, this study found a causal relationship between low mood and empathic distress when viewing other’s pain in healthy participants. This may also have important implications for people with depression, as low mood is one of the key features of depression, and heightened personal distress at seeing others’ pain may result in avoidance and social withdrawal. Further research on this topic, such as the underlying neural activity changes with low mood in response to seeing other’s pain, may help us better understand empathic and emotional processing under low mood, which have implications for better understanding of empathy in depression.

## Author Contributions

YC, RC, and GD had substantial contributions to the design of the work. YC completed the data acquisition and analysis. YC, GC, and RC contributed to the selection of statistical analysis and/or interpretation of the results. YC drafted the paper and GC, RC, and GD revised it critically. All authors approved this final version to be published.

## Conflict of Interest Statement

The authors declare that the research was conducted in the absence of any commercial or financial relationships that could be construed as a potential conflict of interest.
